# The Rosiglitazone-Like Effects of Vitexilactone, a Constituent from *Vitex trifolia* L. in 3T3-L1 Preadipocytes

**DOI:** 10.3390/molecules22112030

**Published:** 2017-11-22

**Authors:** Atsuyoshi Nishina, Masaya Itagaki, Daisuke Sato, Hirokazu Kimura, Yasuaki Hirai, Nyunt Phay, Makoto Makishima

**Affiliations:** 1 College of Science and Technology, Nihon University, 1-5-1 Kandasurugadai, Chiyoda, Tokyo 101-0062, Japan; csms15006@g.nihon-u.ac.jp; 2 Department of Biomedical Information Engineering, Graduate School of Medical Science, Yamagata University, 2-2-2 Iidanishi, Yamagata 990-9585, Japan; d_sato@yz.yamagata-u.ac.jp; 3 School of Medical Technology, Faculty of Health Science, Gunma Paz University, 1-7-1 Tonyamachi, Takasaki, Gunma 370-0006, Japan; h-kimura@paz.ac.jp; 4 Faculty of Arts and Sciences, Showa University, 4562 Kamiyoshida, Fujiyoshida, Yamanashi 403-0005, Japan; hirai@pharm.showa-u.ac.jp; 5 Botany Department, Pathein University, Main Rd., Pathein, Myanmar; dr.nyuntpe@gmail.com; 6 School of Medicine, Nihon University, 30-1 Oyaguchi-kamicho, Itabashi, Tokyo 173-8610, Japan; makishima.makoto@nihon-u.ac.jp

**Keywords:** *Vitex trifolia* L., adipogenesis, lipolysis, lipogenesis, rosiglitazone, vitexilactone

## Abstract

The increased number of patients with type 2 diabetes (T2D) has become a worldwide problem, and insulin sensitizers such as thiazolidinediones (TZDs) are used as therapeutic agents. We found that extracts of *Vitex trifolia* L. (*V. trifolia*), a medicinal plant from Myanmar, induced adipogenesis similar to rosiglitazone (ROS), which is a TZD, in 3T3-L1 preadipocytes. In the present study, we attempted to isolate from *V. trifolia* those compounds that showed ROS-like effects. Among the extracts of hexane, ethyl acetate, and methanol obtained from *V. trifolia*, the ethyl acetate extract with the strongest ROS-like effects was purified by various chromatographic methods to obtain three known compounds: vitexilactone (**1**), vitexicarpin (**2**) and oleanolic acid (**3**). Among the isolated compounds, the ROS-like action of **1** was the strongest. The effects of **1** on 3T3-L1 cells during adipogenesis were compared with those of ROS. Both **1** and ROS increased lipid accumulation, the expression of adiponectin and GLUT4 in the cell membrane and decreased both the size of adipocytes and the phosphorylation of IRS-1, ERK1/2 and JNK in 3T3-L1 cells. In contrast, unlike ROS, the induction of proteins involved in lipogenesis was partial. ROS-like effects of **1** in 3T3-L1 cells were suppressed by the addition of bisphenol A diglycidyl ether (BADGE), one of a peroxisome proliferator-activated receptor γ (PPARγ) antagonists, suggesting that the action of **1** on adipocytes is mediated by PPARγ. From the results of the present study, it can be concluded that **1** is a novel insulin sensitizer candidate.

## 1. Introduction

The number of the patients with type II diabetes (T2D) in the world is expected to reach 300 million in 2025, up from 135 million in 1995 [1]. Thus, T2D represents a serious disease burden. Various hereditary and non-hereditary factors are involved in the development of T2D, but the increase in insulin resistance caused by obesity is the main risk factor [2]. Insulin exhibits multiple physiological effects in humans, such as the down-regulation of blood glucose levels and lipid synthesis of adipocytes. However, the up-regulation of insulin resistance in sugar metabolism is involved in the onset of T2D [3]. Because insulin sensitizers such as thiazolidinediones (TZDs) induce/enhance glucose uptake and metabolism while promoting lipid synthesis and causing weight gain, the development of antidiabetic drugs without lipid synthesis has been desired. TZDs promote adipogenesis by activating peroxisome proliferator activated receptor γ (PPARγ) followed by the down-regulation of insulin resistance [4], but their clinical application is limited to rosiglitazone (ROS) and pioglitazone because of the side effects. However, natural ligands that induce antidiabetic effects via the activation of PPARγ are also being elucidated [5,6,7,8].

The leaves of *Vitex trifolia* L., a traditional medicinal plant from Myanmar, have been reported to be an effective drug for treating amnesia [9], cancer [10,11,12,13], inflammation [14,15], and parasitic infectionw [16], in addition to its wound healing [17] and antibacterial effects [18,19,20]. *V. trifolia* is reported to compounds such as labroane-type diterpenoids [21], terpenoids [11], lignans [22], and flavonoids [13]. We evaluated the effects of leaf extract of *V. trifolia* in 3T3-L1 preadipocytes and found that a constituent, vitexilactone, showed rosiglitazone-like effects. Furthermore, we tried to confirm vitexilactone’s mechanism of action on the rosiglitazone-like effects.

## 2. Results

### 2.1. Yields, Cytotoxicity, and Regulatory Effects on Adipogenesis of the Extracts from V. trifolia

**Figure 1:**
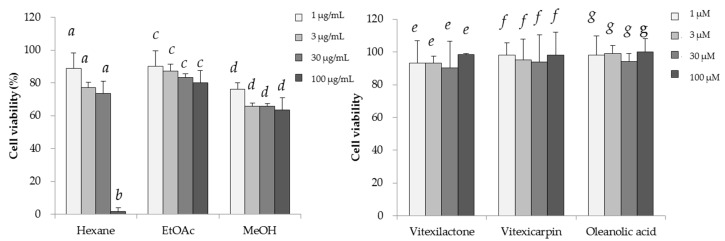
Cytotoxic effects of the three extracts and three compounds isolated from *V. trifolia* in 3T3-L1 cells. Data are expressed as the mean ± SD from three independent experiments. The same letters indicate that there are no differences between those groups, and different letters indicate significant differences (*p* < 0.05).

**Figure 2:**
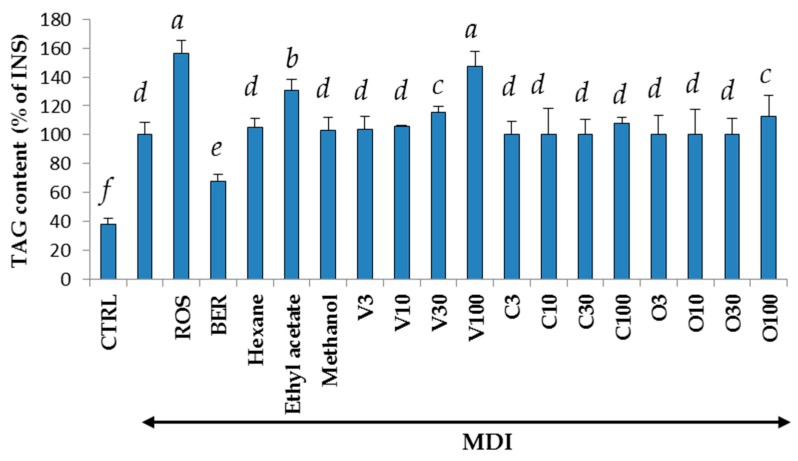
The effects of the three extracts and three compounds isolated from *V. trifolia* on triglycerol levels in 3T3-L1 cells. The 3T3-L1 cells were cultured in 24-well plates and differentiated under the conditions described in the materials and methods section for each compound. Undifferentiated cells, cells with the addition of the MDI mixture (a mixture of 0.5 mM 3-isobutyl-1-methylxanthine (M), 0.1 μM dexamethasone (D), and 2 μM insulin (I)), rosiglitazone, berberine, vitexilactone, vitexicarpin, and oleanolic acid are indicated by CTRL, MDI, ROS, BER, V, C, and O, respectively. Numbers with V, C, and O were concentration (μM) of each compound. On day 8 of culturing, the medium was removed, and cells were lysed using Ripa buffer. The triglycerol levels were determined by the Wako Triglycerol E-test (Wako Pure Chemicals, Osaka, Japan). Data are presented as the mean ± SD from three independent experiments. The same letters indicate that there are no differences between those groups, and different letters indicate significant differences (*p* < 0.05).

Hexane extract (3.9 g), ethyl acetate extract (6.1 g), and methanol extract (3.2 g) were obtained from 100 g of dried leaves of *V. trifolia*. The cytotoxicity and regulation of intracellular triglycerol levels by these extracts are shown in Figure 1 and Figure 2. At a concentration of 30 μg/mL, all of the extracts showed negligible cytotoxicity. Among the three extracts, only the ethyl acetate extract promoted triglycerol accumulation. Thus, the ethyl acetate extract was used to isolate those compounds that promote intracellular triglycerol accumulation in 3T3-L1 cells.

### 2.2. Isolation of Constituents by Chromatography

The ethyl acetate extract (6.1 g) was separated by silica gel column chromatography, eluted with hexane/ethyl acetate (100/0 → 0/100), and divided into four fractions (Fr. A to Fr. D). Due to the fact that Fr. A (2.2 g) was mixture of many trace substances, we could not isolate any of the contained compounds from this fraction. Purification of the main components of Fr. B (265 mg), Fr. C (332 mg), and Fr. D (209 mg) by ODS-HPLC eluted with methanol/water (1/1) afforded compounds **1** (1.5 mg), **2** (9.9 mg), and **3** (3.4 mg).

### 2.3. Characterization of the Isolated Compounds

**Figure 3:**
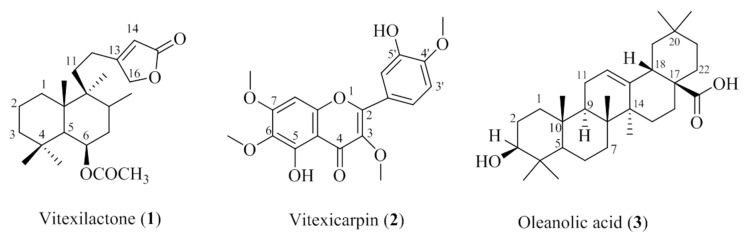
Compounds isolated from *V. trifolia.*

All compounds were identified by comparing their spectral data with the literature (Figure 3). Vitexilactone (**1**) was previously isolated from *Vitex rotundifolia* [23]. Compound **2** displayed flavonoid characteristics in NMR and was identified as vitexicarpin (**2**) [24]. The NMR data of compound **3** possessed the characteristics of a triterpene, and the structure was found to be oleanolic acid (**3**) [25].

### 2.4. Cytotoxicity and Regulatory Effects on Adipogenesis of the Compounds Isolated from V. trifolia

We examined the toxicity of the isolated compounds on 3T3-L1 cells (Figure 1). These compounds did not induce cytotoxicity up to 100 μM. Therefore, the effects of each compound in 3T3-L1 cells were evaluated at 3 to 100 μM under the conditions stated in the Materials and Methods section.

**Figure 4:**
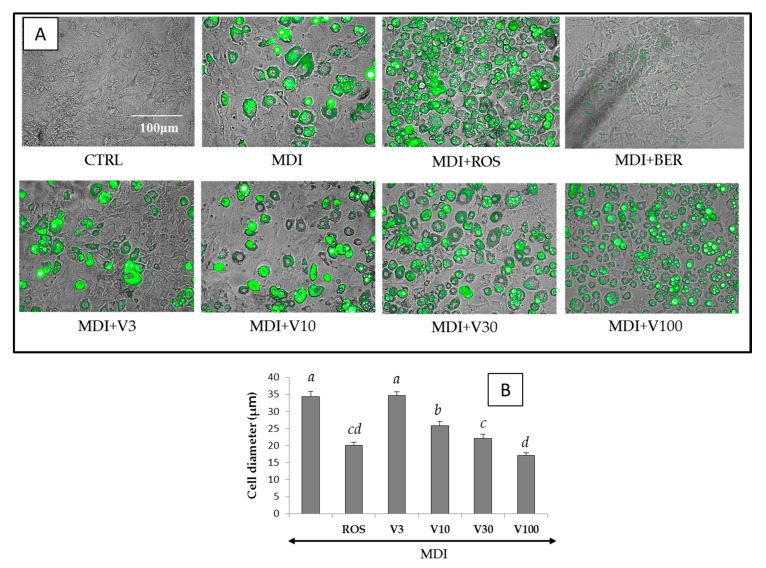
Merged phase differences and fluorescent images and the diameter of differentiated 3T3-L1 cells on day 8 with reference compounds or vitexilactone of various concentrations. The 3T3-L1 cells were cultured in 24-well plates and differentiated with DMI mixture and each compound under the conditions described in the Materials and Methods section. Fluorescent staining of intracellular lipids was accomplished by adding BODIPY 493/503 to the medium. Undifferentiated cells, cells with the addition of MDI (a mixture of 0.5 mM 3-isobutyl-1-methyl xanthine (M), 0.1 μM dexamethasone (D), and 2 μM insulin (I)), rosiglitazone, berberine, and vitexilactone are indicated by CTRL, MDI, ROS, BER, and V, respectively. (**A**) Cell diameters were determined using ImageJ. Data are presented as the mean ± SD from 100 cells of three independent pictures. The same letters indicate that there are no differences between those groups, and different letters indicate significant differences (*p* < 0.05) (**B**).

Differentiation of the 3T3-L1 cells to adipocytes was achieved within 8 days, and the accumulation of intracellular lipids were measured in the presence of compounds **1**–**3** at concentrations of 3 to 100 μM, as described in Section 2.5 (Figure 2). Increased triglyceride accumulation by the extracts or isolated compounds without MDI solution was not significantly different from CTRL (data not shown). When rosiglitazone (ROS) or berberine (BER) with MDI mixture was added to the medium, lipid accumulation increased by 58% and decreased by 33%, respectively. Lipid accumulation increased by 16% or 45% when **1** (30 or 100 μM) or the MDI mixture was added to the medium, respectively, compared with the addition of the MDI mixture only, but the addition of **2** or **3** did not affect the levels of intracellular lipids. Figure 4 shows that the size of the 3T3-L1 cells was dose-dependently miniaturized when **1**, the MDI mixture or ROS was added. Of the three components obtained from *V. trifolia*, only vitexilactone promoted triglycerol accumulation. Thus, vitexilactone was used to confirm the action mechanisms that promote intracellular triglycerol accumulation in 3T3-L1 cells.

### 2.5. The Effects of ***1*** on Insulin Receptor Substrate-1 (IRS-1)

**Figure 5:**
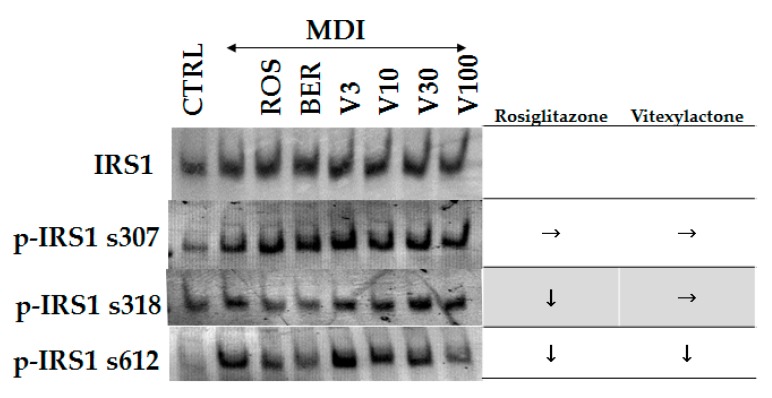
The effects of each compound on the phosphorylation of IRS1 in 3T3-L1 cells during 30 min after the addition of each compound. Protein levels were measured by electroblotting. Undifferentiated cells, cells with the addition of MDI (a mixture of 0.5 mM 3-isobutyl-1-methyl- xanthine (M), 0.1 μM dexamethasone (D), and 2 μM insulin (I)), rosiglitazone, berberine, and vitexilactone are indicated by CTRL, MDI, ROS, BER, and V, respectively. → and ↓ represent no change or down-regulation, respectively.

Binding of insulin to receptors trigger dimerization and autophosphorylation and lead to the initiation of insulin receptor signaling. Tyrosine or serine residues of the IRS located downstream of the insulin receptor are subsequently phosphorylated. It has been reported that the phosphorylation of serine residues of IRS up-regulates insulin resistance [26]. The effect of vitexilactone on IRS-1 is shown in Figure 5. Adding MDI solution to 3T3-L1 cells promoted the phosphorylation of s307, s318, and s612 of IRS-1. ROS and BER inhibited the phosphorylation of s318 and 612 by MDI. On the contrary, vitexilactone only showed a dose-dependent inhibition of phosphorylation of s612 with the MDI mixture.

### 2.6. Effect of Vitexilactone on Signal Transduction-Related Proteins

**Figure 6:**
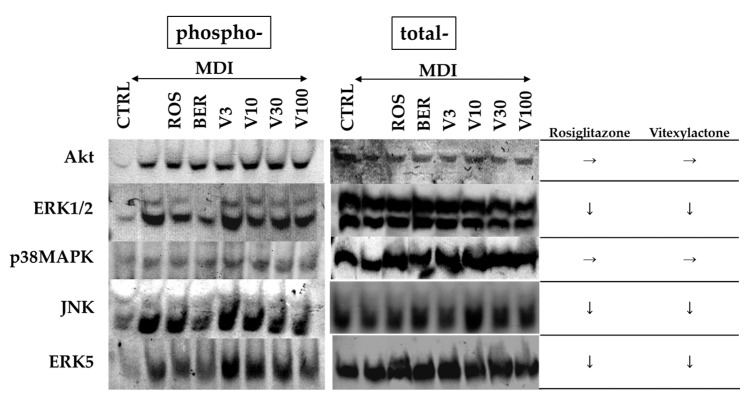
The effects of each compound on the phosphorylation of signal transduction-related kinases in 3T3-L1 cells 30 min after the addition of each compound. Protein levels were measured by electroblotting. Undifferentiated cells, cells with the addition of MDI (a mixture of 0.5 mM 3-isobutyl-1-methylxanthine (M), 0.1 μM dexamethasone (D), and 2 μM insulin (I)), rosiglitazone, berberine, and vitexilactone are indicated by CTRL, MDI, ROS, BER, and V, respectively. → and ↓ represent no change or down-regulation, respectively.

The phosphorylation of IRS in the manner described in Section 2.5 presents various protein binding sites. Among the substances that bind to the protein binding site of phosphorylated IRS, PI3K is a main protein that is involved in the function of insulin via the activation of Akt/PKB and PKCζ cascade [27]. However, insulin signal has been reported to induce growth and cell division through activation of Akt and Ras/MAPK pathway [28]. Although there are many reports on the relationship between insulin signaling and Akt, there are relatively few reports on the involvement of MAPK in adipogenesis. The levels of phosphorylation of Akt and MAPKs at 30 min after addition of MDI solution and test compounds to the medium are shown in Figure 6. Phosphorylation of Akt, ERK1/2, JNK, and ERK5 was promoted by the addition of MDI solution. ROS and BER inhibited the MDI solution-induced phosphorylation of ERK1/2, JNK, and ERK5. The phosphorylation suppression was higher for ROS than for BER. In contrast, vitexilactone suppressed the phosphorylation of ERK1/2, JNK, and ERK5 in a dose-dependent manner.

### 2.7. Effect of Vitexilactone on Sugar/Fat Metabolism-Related Proteins

**Figure 7:**
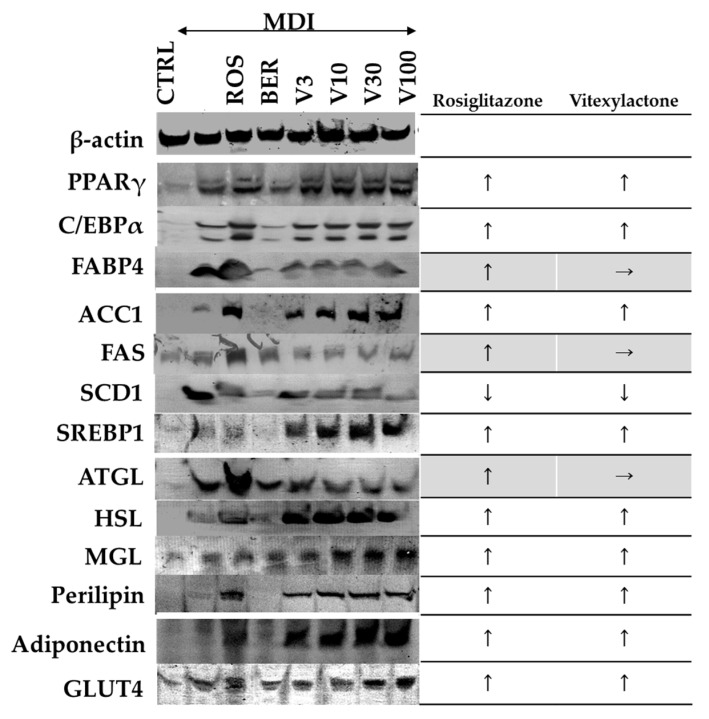
The effects of each compound on adipogenesis-related proteins, adiponectin, and, GLUT4 levels in 3T3-L1 cells on day 8. The 3T3-L1 cells were cultured in 6-well plates and differentiated with DMI mixture with or without each compound under the conditions described in the Materials and Methods section and subsequently lysed with lysis buffer. Cell lysates were collected using a cell scraper and centrifuged at 15,000× *g* for 30 min at 4 °C. The supernatant was collected and the overall protein concentration was determined by a Protein Assay Reagent Kit (Cytoskeleton, Denver, CO, USA) with BSA as the standard. To detect GLUT4, membrane protein was extracted using Plasma Membrane Protein Extraction Kit according to the instructions of the manufacturer. Protein levels were measured by electroblotting. →, ↑ and ↓ represent no change, up- or down-regulation, respectively.

CCAAT/enhancer-binding protein α (C/EBPα), peroxisome proliferator-activated receptor (PPARγ), and fatty-acid-binding protein4 (FABP4) (adipogenic factors), acetyl-CoA carboxylase 1 (ACC1), fatty acid synthase (FAS), stearoyl-CoA desaturase-1 (SCD-1), and sterol regulatory element-binding protein-1 (SREBP-1) (lipogenic factors), adipose triglyceride lipase (ATGL), hormone-sensitive lipase (HSL), monoacylglycerol lipase (MGL), and perilipin (lipolytic factoros), adiponectin, and glucose transporter type 4 (GLUT4) in the cell membrane along with adipogenesis (day eight) are shown in Figure 7.

Adding MDI solution to the medium resulted in increased expression levels of all 12 proteins tested. Adding MDI solution in combination with ROS resulted in a further increase in the expression levels of all proteins, except SCD1 (SREBP1 and MGL expression was slightly increased by ROS). In comparison, adding MDI solution with vitexilactone resulted in increased expression levels of all proteins, except FABP4, FAS and ATGL, at levels comparable to treatment with MDI solution and ROS. Vitexilactone-induced proteins related to adipogenesis and lipolysis in the same manner as ROS, but its effect on the levels on the expression of lipogenic proteins was limited.

### 2.8. Effect of PPARγ Antagonist and MAPKs Inhibitor on Intracellular Lipid Accumulation by MDI Solution with or without ROS or Vitexilactone

**Figure 8:**
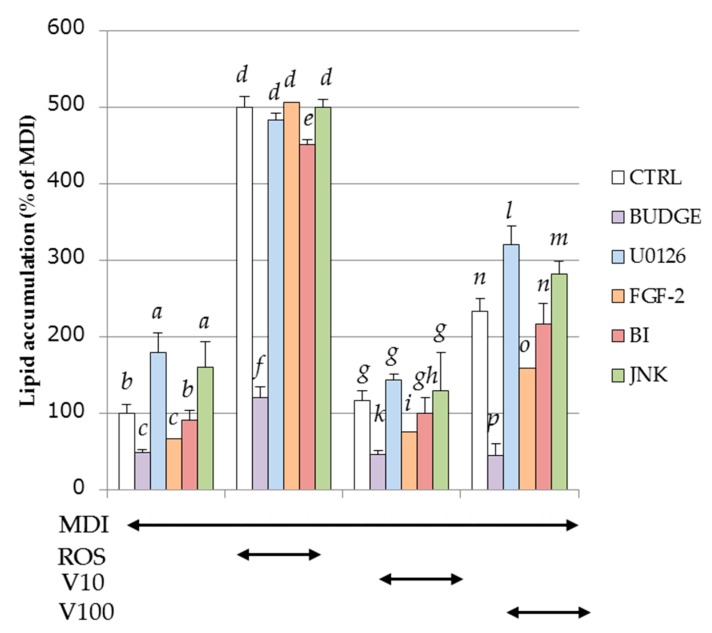
The effects of each inhibitor on triglycerol levels in 3T3-L1 cells during adipogenesis. Cells were treated with the condition described in the text. On day 8 of culturing, the triglycerol levels were determined by the Wako Triglycerol E-test (Wako). Data are presented as the mean ± SD from three independent experiments. The same letters indicate that there are no differences between those groups, and different letters indicate significant differences (*p* < 0.05). Undifferentiated cells, cells with the addition of MDI (a mixture of 0.5 mM 3-isobutyl-1-methylxanthine (M), 0.1 μM dexamethasone (D), and 2 μM insulin (I)), rosiglitazone, vitexilactone, BIX02188, and JNK inhibitor are indicated by CTRL, MDI, ROS, V, BI and, JNK, respectively.

The effects of PPARγ antagonist and MAPKs inhibitor on intracellular lipid accumulation during adipogenesis by MDI solution, with or without ROS or vitexilactone, are shown in Figure 8. BADGE (an antagonist of PPARγ) decreased lipid accumulation to 24%, 40%, and 19% in MDI plus ROS, vitexilactone (10 μM), and MDI plus vitexilactone (100 μM), respectively, while it did to 47% in MDI alone (Figure 8), suggesting that vitexilactone activates PPARγ similar to ROS. The accumulation of intracellular lipids by MDI solution with ROS was not affected by various MAPK inhibitors, which suggests that ROS and MAPKs may be unrelated to intracellular lipid accumulation. In contrast, intracellular lipid accumulation was increased by U0126 (MEK1/2 inhibitor) and JNK inhibitor and decreased by FGF-2 (MAPK phosphorylation enhancer). Therefore, it was deduced that vitexilactone up-regulated intracellular lipid accumulation by inhibition of phosphorylation of MEK1/2, followed by ERK1/2, and JNK. In addition, it was presumed that the phosphorylation of ERK5 and intracellular lipid accumulation were unrelated because the addition of BIX02188 (ERK5 inhibitor) and cellular lipid accumulation were unrelated.

## 3. Discussion

Insulin resistance is induced by phosphorylation of serine residues (Ser 307/612/632) of IRS-1 by diabetes-inducing factors such as fatty acids, and TNFα [29]. As vitexilactone suppressed the phosphorylation of serine residues of IRS-1 such as rosiglitagone (Figure 5), vitexilactone may improve insulin sensitivity.

Due to insulin stimulation, insulin receptor, IRS, PI3K, and Akt were phosphorylated in this order, and glucose uptake was promoted by the migration of GLUT4 to the cell membrane [30]. In this study, ROS and vitexilactone increased GLUT4 in the cell membrane (Figure 6), although ROS and vitexilactone showed no effect on the promotion of Akt phosphorylation by MDI solution (Figure 7). Therefore, pathways for transferring GLUT4 to the cell membrane independent of IRS/Akt pathway may exist.

Moreover, we found that both ROS and vitexilactone suppressed the phosphorylation levels of ERK1/2, JNK, and ERK 5 promoted by MDI solution (Figure 6). In contrast, the lipid accumulation of 3T3-L1 cells promoted by MDI solution with vitexilactone was down-regulated by MEK1/2 inhibitor (U0126) and JNK inhibitor and up-regulated by FGF-2, an inducer of MAPK phosphorylation (Figure 8). Thus, vitexilactone is considered to promote the lipid accumulation of 3T3-L1 cells during adipogenesis by inhibiting the phosphorylation of ERK1/2 and/or JNK. Since the lipid accumulation of 3T3-L1 cells promoted by MDI solution and ROS was unaffected by U0126, JNK inhibitor, and FGF-2, the relationship between lipid accumulation by ROS and phosphorylation of MAPK remains unclear. The addition of ERK5 inhibitor (BIX 02188) showed no change in lipid accumulation in 3T3-L1 cells promoted by MDI solution, suggesting that ERK5 phosphorylation and lipid accumulation are unrelated.

Among the adipogenesis-related proteins (C/EBPα, PPARγ, and FABP4), both vitexilactone and ROS up-regulated the expression levels of C/EBPα and PPARγ at comparable levels. However, unlike ROS, vitexilactone did not up-regulate the expression of FABP4. In the present study, the expression level of FABP4 was checked as an indicator of adipogenesis. FABP4 is also known to induce proteasome degradation of PPARγ, a master regulator of lipid production and insulin response via ubiquitination [31]. Therefore, it may be possible for vitexilactone to increase the expression level of PPARγ without increasing FABP4.

ROS up-regulated the expression of lipogenic proteins (ACC1, FAS, SCD1, and SREBP1). However, vitexilactone dose-dependently up-regulated the expression of ACC1, SCD1, and SREBP1 but not FAS. FAS is one of the main enzymes that increase lipid accumulation in adipocytes, and it was reported that the expression level of FAS correlates with obesity [32]. Since one of the side effects of ROS is weight gain, vitexilactone may mitigate the weight gain effects of ROS.

ROS increased the expression of lipolytic proteins (ATGL, HSL, MGL, and perilipin), but vitexilactone enhanced the expression of only HSL, MGL, and perilipin dose-dependently. An earlier report showed that ATGL decreases with insulin stimulation [33]. Nevertheless, in the experimental system of this study, the expression of ATGL was promoted by MDI solution, with a further increase in expression level when ROS was also added. ATGL is one of the enzymes responsible for lipid metabolism together with HSL, and an up-regulation of the expression level of ATGL increases insulin resistance and insulin level in the blood [34]. Since vitexilactone did not increase the expression level of ATGL, it may be considered more advantageous than ROS in terms of insulin resistance.

The functions of adiponectin include promoting glucose uptake without activating insulin receptor, metabolizing fatty acid, reducing the fatty acid content in the cell by enhancing insulin receptor sensitivity, activating liver AMP kinase, inhibiting arteriosclerosis, exerting anti-inflammation activity, and suppressing myocardial hypertrophy [35]. Furthermore, it has been recognized that adiponectin secretion is up-regulated during the miniaturization of hypertrophic adipocytes [36]. Both ROS and vitexilactone induced the miniaturization of adipocytes during adipogenesis (Figure 4) and increased the secretion of adiponectin, suggesting that vitexilactone may possess antidiabetic effects.

Since the lipid accumulation of 3T3-L1 cells promoted by ROS or vitexilactone with MDI solution was inhibited by PPARγ antagonist (BADGE), vitexilactone may induce adipogenesis as an agonist of PPARγ, similar to ROS. Vitexilactone did not induce the expression of the PPARγ target FABP4 (Figure 7). Further studies, including transactivation assay and structural analysis for ligand interaction, are needed to elucidate the molecular mechanisms by which vitexilactone exerts partial ROS-like effects in adipocytes. Meanwhile, inhibitors of phosphorylation of MEK1/2 or JNK promoted lipid accumulation, but the activator of intracellular MAPKs decreased lipid accumulation, suggesting that the suppression of MAPK phosphorylation plays a major role in the promotion of intracellular lipid accumulation by vitexilactone.

In the present study, it was confirmed that effects of vitexilactone and ROS in 3T3-L1 cells during adipogenesis were very similar. However, unlike ROS, vitexilactone did not promote the expression of FABP4, FAS (promoter of obesity), or ATGL (inducer of insulin resistance). The above mentioned results indicate that vitexilactone is a promising drug discovery lead candidate for improving diabetes and has many benefits over ROS in terms of anti-obesity.

## 4. Materials and Methods

### 4.1. Reagents

Hexane, ethyl acetate, and methanol were obtained from Wako Pure Chemical Industries (Osaka, Japan). A mitogen-activated protein kinase (MAPK)/ERK kinase (MEK) inhibitor (U0126), a c-Jun N-terminal kinase (JNK) inhibitor, and an extracellular signal-regulated kinase 5 (ERK5) inhibitor (BIX02188) were purchased from Calbiochem (San Diego, CA, USA). Fibroblast growth factor-2 (FGF-2) and a peroxisome proliferator-activated receptor γ (PPARγ) inhibitor (Bisphenol A diglycidyl ether (BUDGE)) were obtained from Peprotech (Rocky Hill, NJ, USA) and TCI (Tokyo, Japan), respectively.

### 4.2. Solvent Fractionation

Dried leaves of *Vitex trifolia* L. was purchased commercially from an herbal medicine market in Yangon, Myanmar and identified by Dr. Nyunt Phay (Rector, Pathein University, Pathein, Myanmar). Powder of *V. trifolia* leaves (100 g) was immersed in hexane (500 mL) for 24 h at room temperature. The solvent containing the extracts was filtrated through a filter paper (5C; Whatman, Brentford, UK) and the filtrate was evaporated to dryness (hexane extract; 3.9 g). The residue was then stirred in 500 mL of ethyl acetate at room temperature for 24 h and filtrated, and the filtrate was evaporated to dryness (ethyl acetate extract; 6.1 g). The methanol extract (3.2 g) was prepared in a similar manner.

### 4.3. Isolation of Active Constituents

The ethyl acetate fraction (6.1 g) was divided by silica gel column chromatography (CC) eluted with hexane/ethyl acetate (1/0 to 0/1; *v*/*v*), to give four fractions (Fr. A to Fr. D). Separation of Fr. B (265 mg), C (332 mg), and D (209 mg) by HPLC equipped with an ODS column eluting with MeOH/water = 1/1 afforded compound **1** (1.5 mg), **2** (9.9 mg), and **3** (3.4 mg), respectively. Purity of compounds **1**–**3** was confirmed as more than 95% by measurement of the ^1^H-NMR spectra with dimethyl sulfone as the internal standard [37].

### 4.4. Analytical Instrument of Isolated Components

^1^H (400 MHz) and ^13^C (100 MHz) NMR spectra were recorded with a ECX 400 spectrometer (JEOL, Tokyo, Japan) with tetramethylsilane as an internal standard. The high-resolution MS were obtained using a LC/MSD TOF-G1969A system (Agilent Technologies, Santa Clara, CA, USA).

### 4.5. Cell Culture

Murine 3T3-L1 preadipocytes were propagated in the Dulbecco’s Modified Eagle medium (DMEM) supplemented with 10% calf serum until 80% confluence (day 0) and the medium was replaced with DMEM containing a 10% FBS, MDI mixture (a mixture of 0.5 mM 3-isobutyl-1-methyl xanthine (M), 0.1 μM dexamethasone (D) and, 2 μM insulin (I)), with or without one of a test compound. After 48 h (day 2), the medium was replaced with DMEM containing 10% FBS and 2 μM insulin. After 48 h (day 4), the medium was replaced with DMEM containing 10% FBS. Thereafter, the medium was exchanged every other day [38]. 0.1 μM or 2.7 nM of ROS or BER was used as positive or negative reference compound, respectively. Cells were maintained in a humidified atmosphere of 5% CO_2_ at 37 °C.

### 4.6. Cell Toxicity Assay

3T3-L1 cells were seeded in 96-well plate with DMEM supplemented with 10% calf serum until 80% confluence and the medium was replaced with DMEM containing a 10% FBS with or without one of a test compound. Thereafter, the medium without test compounds was exchanged every other day. Cytotoxicity was measured by the use of Cell Counting Kit-8 (Dojindo, Kumamoto, Japan) according to the instructions of the manufacturer. Absorbance was measured at 450 nm by using Sunrise Absorbance Reader (Tecan, Männedorf, Switzerland).

### 4.7. Measurement of Intracellular Triglycerol Level

Intracellular triglycerol levels in 3T3-L1 cells at day 8 were measured by using of E-test WAKO Triglyceride Kit (Wako Pure Chemical) according to the instructions of the manufacturer. 100 ng/mL of BODIPY 493/503 were added to culture medium followed by incubation of 10 min. Images were taken by a fluorescent cell imager (Floid Cell Imaging Solution; Life Technologies, Carlsbad, CA, USA) [38].

### 4.8. Detection of Proteins

Differentiated (day 8) 3T3-L1 cells in 6-well plates were placed on ice and each well was washed with PBS, and subsequently lysed with 150 μL of 20 mM Tris-HCl buffer (pH 8.0) containing 150 mM NaCl, 2 mM EDTA, 1% Nonidet P-40 (*w*/*v*), 1% sodium deoxycholate (*w*/*v*), 0.1% sodium dodecyl sulfate (*w*/*v*), 50 mM NaF, 0.1% aprotinin (*w*/*v*), 0.1% leupeptin (*w*/*v*), 1 mM Na_3_VO_4_, and 1 mM phenylmethylsulphonylfluoride (PMSF). Cell lysates were collected using a cell scraper and centrifuged at 15,000× *g* for 30 min at 4 °C. The supernatant was collected and the overall protein concentration was determined by a Protein Assay Reagent Kit (Cytoskeleton, Denver, CO, USA) with BSA as the standard. To detect GLUT4, membrane protein was extracted using a Plasma Membrane Protein Extraction Kit (101 Bio, Palo Alto, CA, USA) according to the instructions of the manufacturer.

Supernatant fluids containing proteins were mixed with lithium dodecyl sulfate (LDS) sample buffer (Invitrogen Corp, Carlsbad, CA, USA) and incubated for 5 min at 80 °C. Samples containing proteins (20 μg) were loaded in each lane followed by separation on SDS-polyacrylamide gel electrophoresis, and the proteins in gels were electroblotted onto polyvinylidene fluoride (PVDF) filters (Hybond-P, 0.2 μm; GE Healthcare, Little Chalfont, UK). Immunoblotting analysis was performed by using monoclonal antibodies against insulin receptor substrate-1 (IRS-1) (180 kDa), phospho-IRS-1 (s307, 318, and 612) (180 kDa), Akt (60 kDa), phospho-Akt (60 kDa), extracellular signal-regulated kinase1/2 (ERK1/2) (42 and 44 kDa), phospho-ERK1/2 (42 and 44 kDa), p38 mitogen-activated protein kinase (p38MAPK) (40 kDa), phospho-p38MAPK (40 kDa), c-Jun-NH_2_-terminal kinase (JNK) (46 and 54 kDa), phospho-JNK (46 and 54 kDa), ERK5 (115 kDa), phospho-ERK5 (115 kDa), acetyl-CoA carboxylase (ACC) (280 kDa), β-actin (45 kDa), CCAAT/enhancer-binding protein α (C/EBPα) (42 kDa), fatty-acid-binding protein4 (FABP4) (15 kDa), peroxisome proliferator-activated receptor (PPARγ) (53 and 57 kDa), fatty acid synthase (FAS) (273 kDa), stearoyl-CoA desaturase-1 (SCD-1) (42 kDa), adipose triglyceride lipase (ATGL) (54 kDa), hormone-sensitive lipase (HSL) (81 and 83 kDa), perilipin (62 kDa), adioponectin (27 kDa), and glucose transporter type 4 (GLUT4) (50 kDa) (Cell Signaling Technology, Lake Placid, NY, USA), sterol regulatory element-binding protein-1 (SREBP-1) (114 kDa) and monoacylglycerol lipase (MGL) (33 kDa) (Santa Cruz Biotechnology, Dallas, TX, USA) as the primary antibodies, followed by reaction with horseradish peroxidase-conjugated anti-rabbit IgG antibodies from Sigma-Aldrich (St. Louis, MO, USA) as the secondary antibody. Primary and secondary antibodies were diluted 1000 or 3000 times for use, respectively. The blots were developed by the enhanced chemiluminescence method (Western Lightning ECL Pro; Perkin Elmer, Waltham, MA, USA) [39]. Three independent experiments were performed and densitometric intensity and SD measured with ImageJ are listed in Tables D–F of the Supplementary Material file.

### 4.9. Treatment with Specific Inhibitors

3T3-L1 cells were seeded in 24-well plate with DMEM supplemented with 10% calf serum until 80% confluence (day 0) and each inhibitor was added to medium to result in a final concentration of 100 μM (BUDGE), 30 μM (U0126, BIX02188, JNK inhibitor), and 6 μM (FGF-2) for 1 h. Cells were then differentiated in the condition described in Section 4.5. 

### 4.10. Statistical Analysis

The results were expressed as mean ± standard deviation (SD). The significant difference between the groups compared were determined using analysis of variance (ANOVA) followed by Tukey test.

## 5. Conclusions

We isolated and identified three compounds, vitexilactone (**1**), vitexicarpin (**2**) and oleanolic acid (**3**), from the ethyl acetate extract of *Vitex trifolia* L., which up-regulates lipid accumulation and adipogenesis in 3T3-L1 preadipocytes. Among the isolated compounds, the rosiglitazone (ROS)-like effects of **1** were the strongest. Compound **1** and ROS increased lipid accumulation and the expression of adiponectin and GLUT4 in the cell membrane and miniaturized the adipocytes and decreased the phosphorylation of IRS-1, ERK1/2 and JNK in 3T3-L1 cells. Since ROS-like effects of **1** in 3T3-L1 cells was suppressed by the addition of bisphenol A diglycidyl ether (BADGE), a peroxisome proliferator-activated receptor γ (PPARγ) antagonist, **1** was considered to act on adipocytes as an action selective PPARγ agonist. From the results in the present study, **1** is a potential candidate for use as a novel insulin sensitizer.

## Supplementary Materials

The following are available online.
