# Supplementary material for: The Rosiglitazone-Like Effects of Vitexilactone, a Constituent from Vitex trifolia L. in 3T3-L1 Preadipocytes
Source: Molecules. 2017 Nov 22;22(11):2030. doi: 10.3390/molecules22112030 (PMC6150318; doi:10.3390/molecules22112030)
Supplement: Supplementary file 1 [file molecules-22-02030-s001.pptx]

## Slide 1
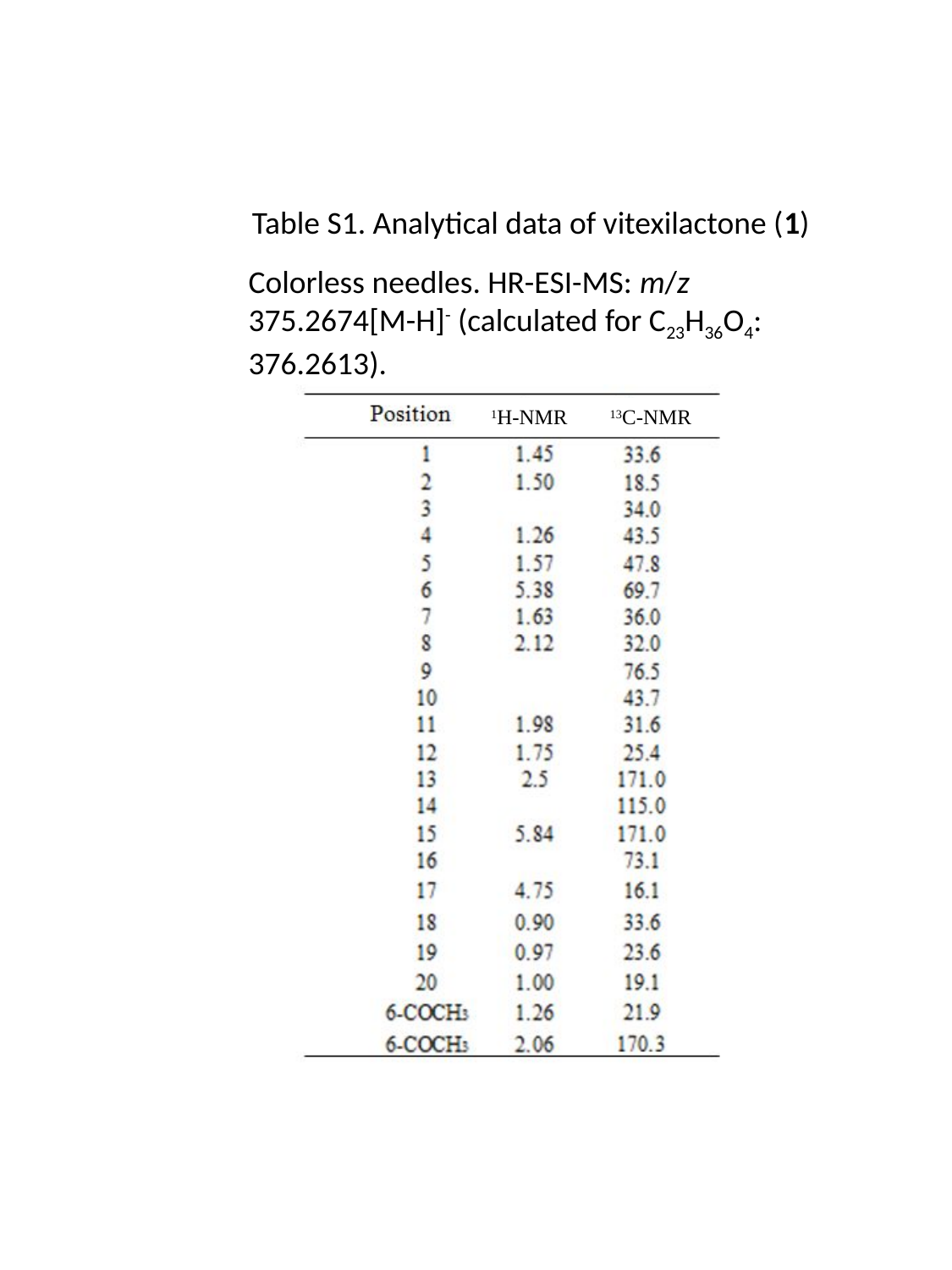

Table S1. Analytical data of vitexilactone (1)
Colorless needles. HR-ESI-MS: m/z 375.2674[M-H]- (calculated for C23H36O4: 376.2613).
1H-NMR
13C-NMR

## Slide 2
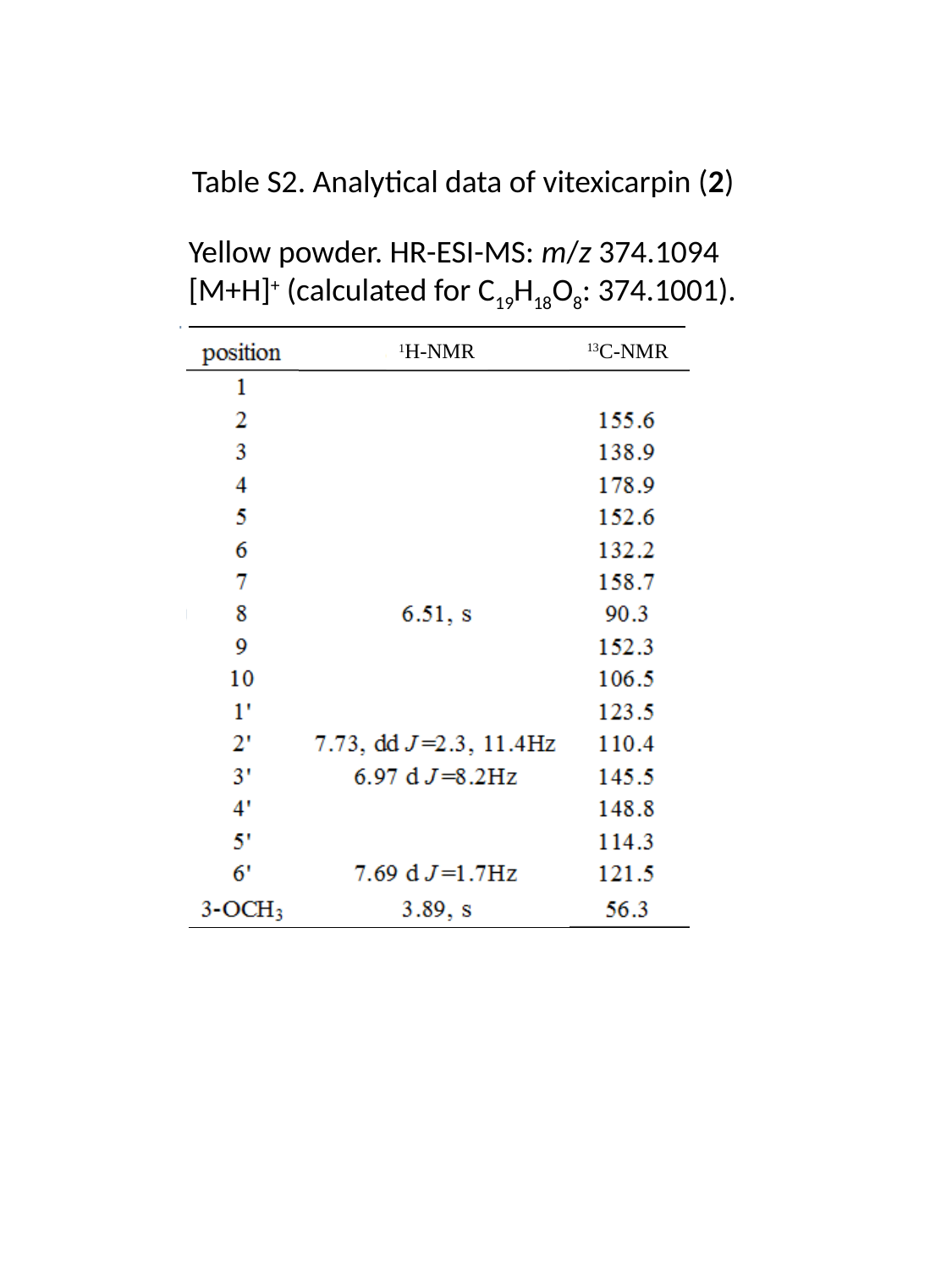

Table S2. Analytical data of vitexicarpin (2)
Yellow powder. HR-ESI-MS: m/z 374.1094 [M+H]+ (calculated for C19H18O8: 374.1001).
13C-NMR
1H-NMR

## Slide 3
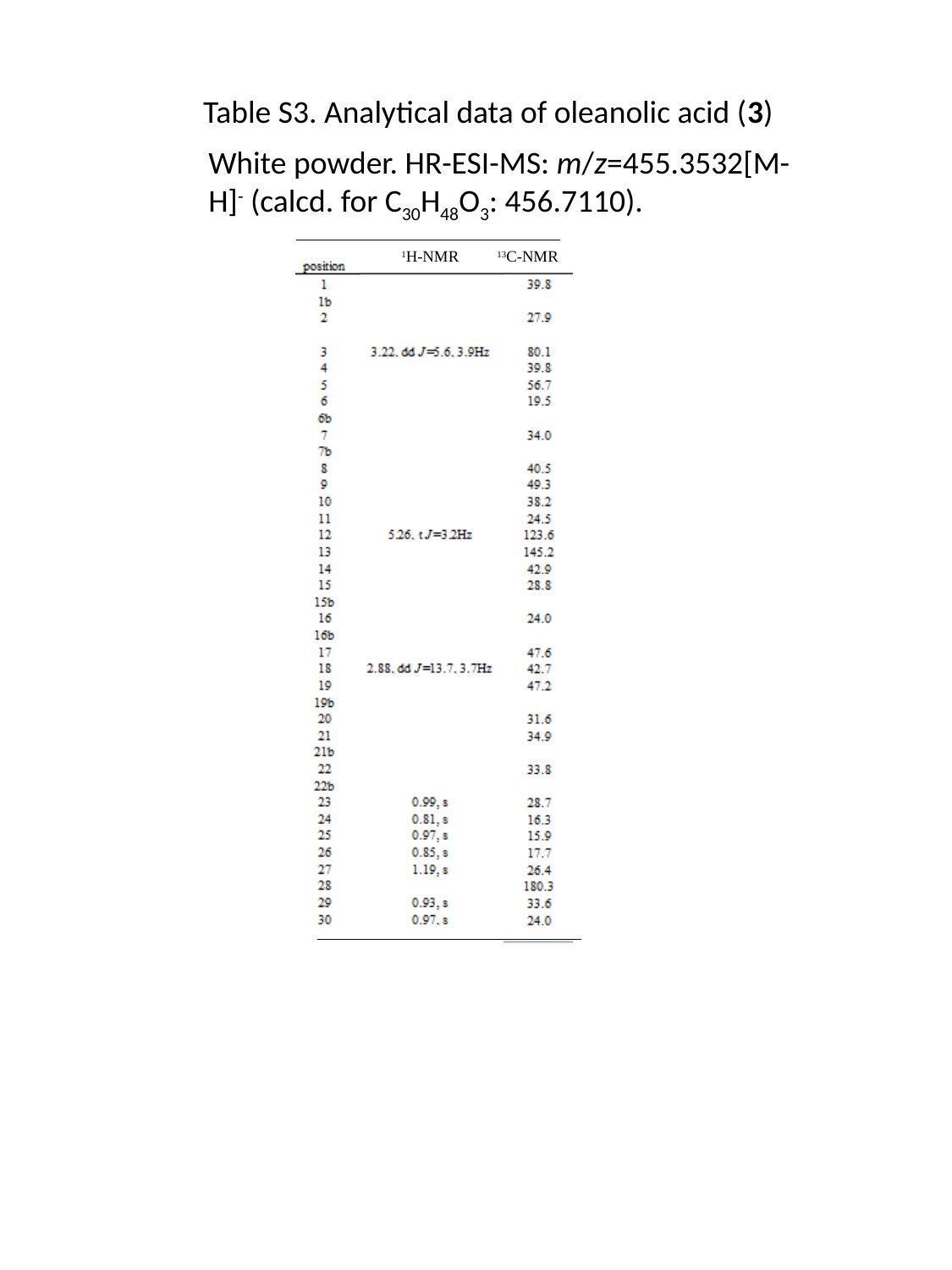

Table S3. Analytical data of oleanolic acid (3)
White powder. HR-ESI-MS: m/z=455.3532[M-H]- (calcd. for C30H48O3: 456.7110).
1H-NMR
13C-NMR

## Slide 4
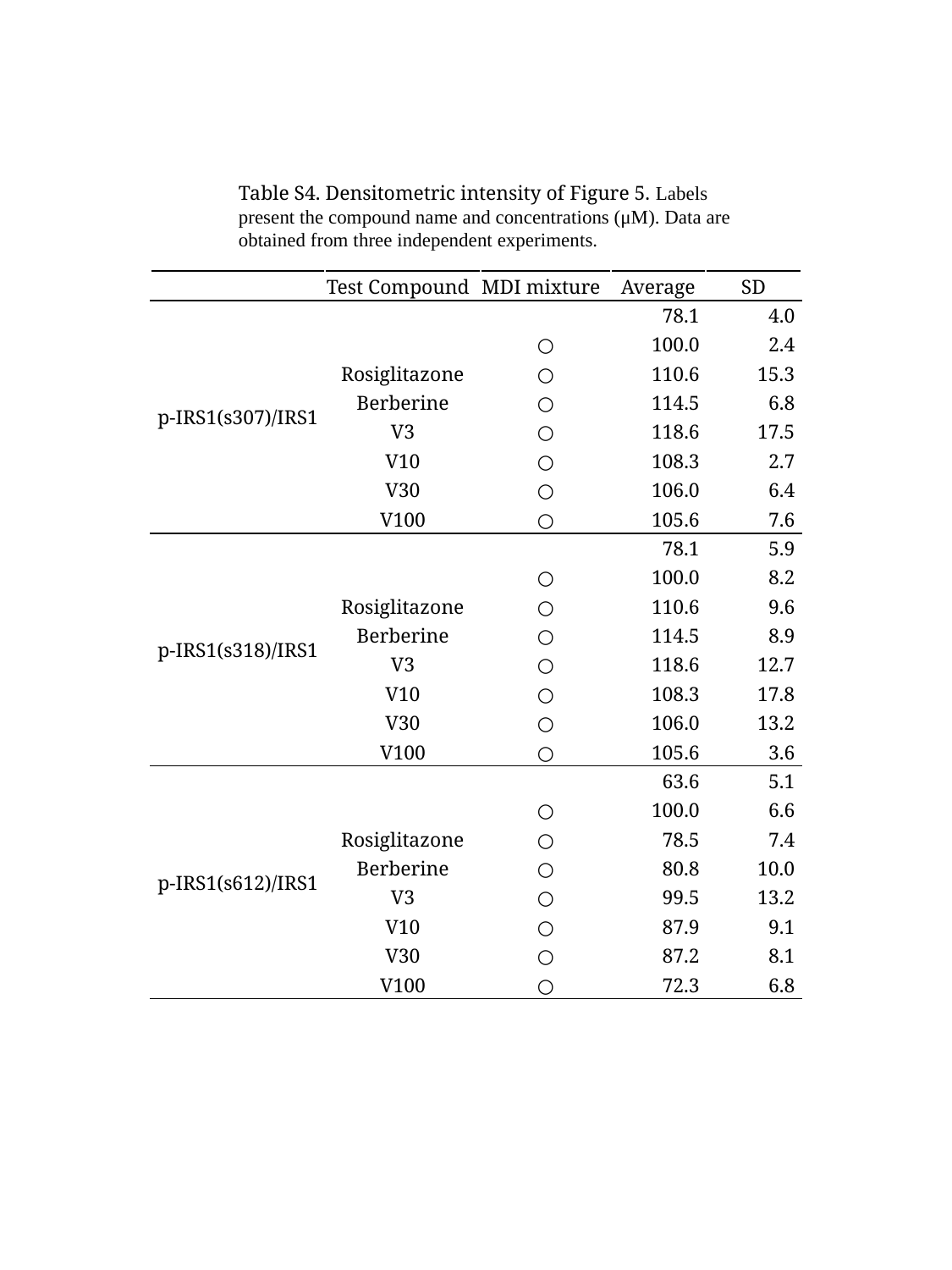

Table S4. Densitometric intensity of Figure 5. Labels present the compound name and concentrations (μM). Data are obtained from three independent experiments.
| | Test Compound | MDI mixture | Average | SD |
| --- | --- | --- | --- | --- |
| p-IRS1(s307)/IRS1 | | | 78.1 | 4.0 |
| | | ○ | 100.0 | 2.4 |
| | Rosiglitazone | ○ | 110.6 | 15.3 |
| | Berberine | ○ | 114.5 | 6.8 |
| | V3 | ○ | 118.6 | 17.5 |
| | V10 | ○ | 108.3 | 2.7 |
| | V30 | ○ | 106.0 | 6.4 |
| | V100 | ○ | 105.6 | 7.6 |
| p-IRS1(s318)/IRS1 | | | 78.1 | 5.9 |
| | | ○ | 100.0 | 8.2 |
| | Rosiglitazone | ○ | 110.6 | 9.6 |
| | Berberine | ○ | 114.5 | 8.9 |
| | V3 | ○ | 118.6 | 12.7 |
| | V10 | ○ | 108.3 | 17.8 |
| | V30 | ○ | 106.0 | 13.2 |
| | V100 | ○ | 105.6 | 3.6 |
| p-IRS1(s612)/IRS1 | | | 63.6 | 5.1 |
| | | ○ | 100.0 | 6.6 |
| | Rosiglitazone | ○ | 78.5 | 7.4 |
| | Berberine | ○ | 80.8 | 10.0 |
| | V3 | ○ | 99.5 | 13.2 |
| | V10 | ○ | 87.9 | 9.1 |
| | V30 | ○ | 87.2 | 8.1 |
| | V100 | ○ | 72.3 | 6.8 |

## Slide 5
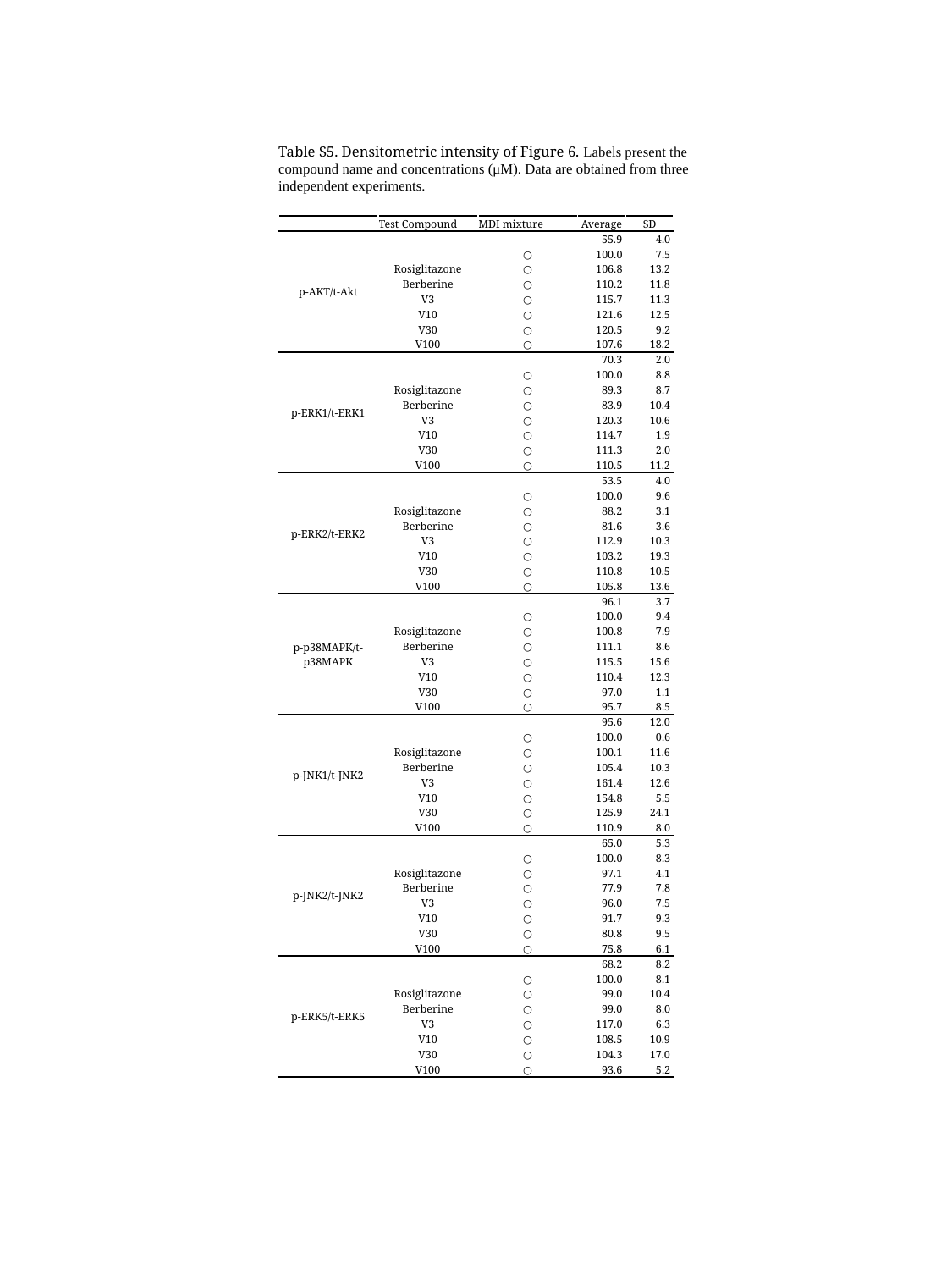

Table S5. Densitometric intensity of Figure 6. Labels present the compound name and concentrations (μM). Data are obtained from three independent experiments.
| | Test Compound | MDI mixture | Average | SD |
| --- | --- | --- | --- | --- |
| p-AKT/t-Akt | | | 55.9 | 4.0 |
| | | ○ | 100.0 | 7.5 |
| | Rosiglitazone | ○ | 106.8 | 13.2 |
| | Berberine | ○ | 110.2 | 11.8 |
| | V3 | ○ | 115.7 | 11.3 |
| | V10 | ○ | 121.6 | 12.5 |
| | V30 | ○ | 120.5 | 9.2 |
| | V100 | ○ | 107.6 | 18.2 |
| p-ERK1/t-ERK1 | | | 70.3 | 2.0 |
| | | ○ | 100.0 | 8.8 |
| | Rosiglitazone | ○ | 89.3 | 8.7 |
| | Berberine | ○ | 83.9 | 10.4 |
| | V3 | ○ | 120.3 | 10.6 |
| | V10 | ○ | 114.7 | 1.9 |
| | V30 | ○ | 111.3 | 2.0 |
| | V100 | ○ | 110.5 | 11.2 |
| p-ERK2/t-ERK2 | | | 53.5 | 4.0 |
| | | ○ | 100.0 | 9.6 |
| | Rosiglitazone | ○ | 88.2 | 3.1 |
| | Berberine | ○ | 81.6 | 3.6 |
| | V3 | ○ | 112.9 | 10.3 |
| | V10 | ○ | 103.2 | 19.3 |
| | V30 | ○ | 110.8 | 10.5 |
| | V100 | ○ | 105.8 | 13.6 |
| p-p38MAPK/t-p38MAPK | | | 96.1 | 3.7 |
| | | ○ | 100.0 | 9.4 |
| | Rosiglitazone | ○ | 100.8 | 7.9 |
| | Berberine | ○ | 111.1 | 8.6 |
| | V3 | ○ | 115.5 | 15.6 |
| | V10 | ○ | 110.4 | 12.3 |
| | V30 | ○ | 97.0 | 1.1 |
| | V100 | ○ | 95.7 | 8.5 |
| p-JNK1/t-JNK2 | | | 95.6 | 12.0 |
| | | ○ | 100.0 | 0.6 |
| | Rosiglitazone | ○ | 100.1 | 11.6 |
| | Berberine | ○ | 105.4 | 10.3 |
| | V3 | ○ | 161.4 | 12.6 |
| | V10 | ○ | 154.8 | 5.5 |
| | V30 | ○ | 125.9 | 24.1 |
| | V100 | ○ | 110.9 | 8.0 |
| p-JNK2/t-JNK2 | | | 65.0 | 5.3 |
| | | ○ | 100.0 | 8.3 |
| | Rosiglitazone | ○ | 97.1 | 4.1 |
| | Berberine | ○ | 77.9 | 7.8 |
| | V3 | ○ | 96.0 | 7.5 |
| | V10 | ○ | 91.7 | 9.3 |
| | V30 | ○ | 80.8 | 9.5 |
| | V100 | ○ | 75.8 | 6.1 |
| p-ERK5/t-ERK5 | | | 68.2 | 8.2 |
| | | ○ | 100.0 | 8.1 |
| | Rosiglitazone | ○ | 99.0 | 10.4 |
| | Berberine | ○ | 99.0 | 8.0 |
| | V3 | ○ | 117.0 | 6.3 |
| | V10 | ○ | 108.5 | 10.9 |
| | V30 | ○ | 104.3 | 17.0 |
| | V100 | ○ | 93.6 | 5.2 |

## Slide 6
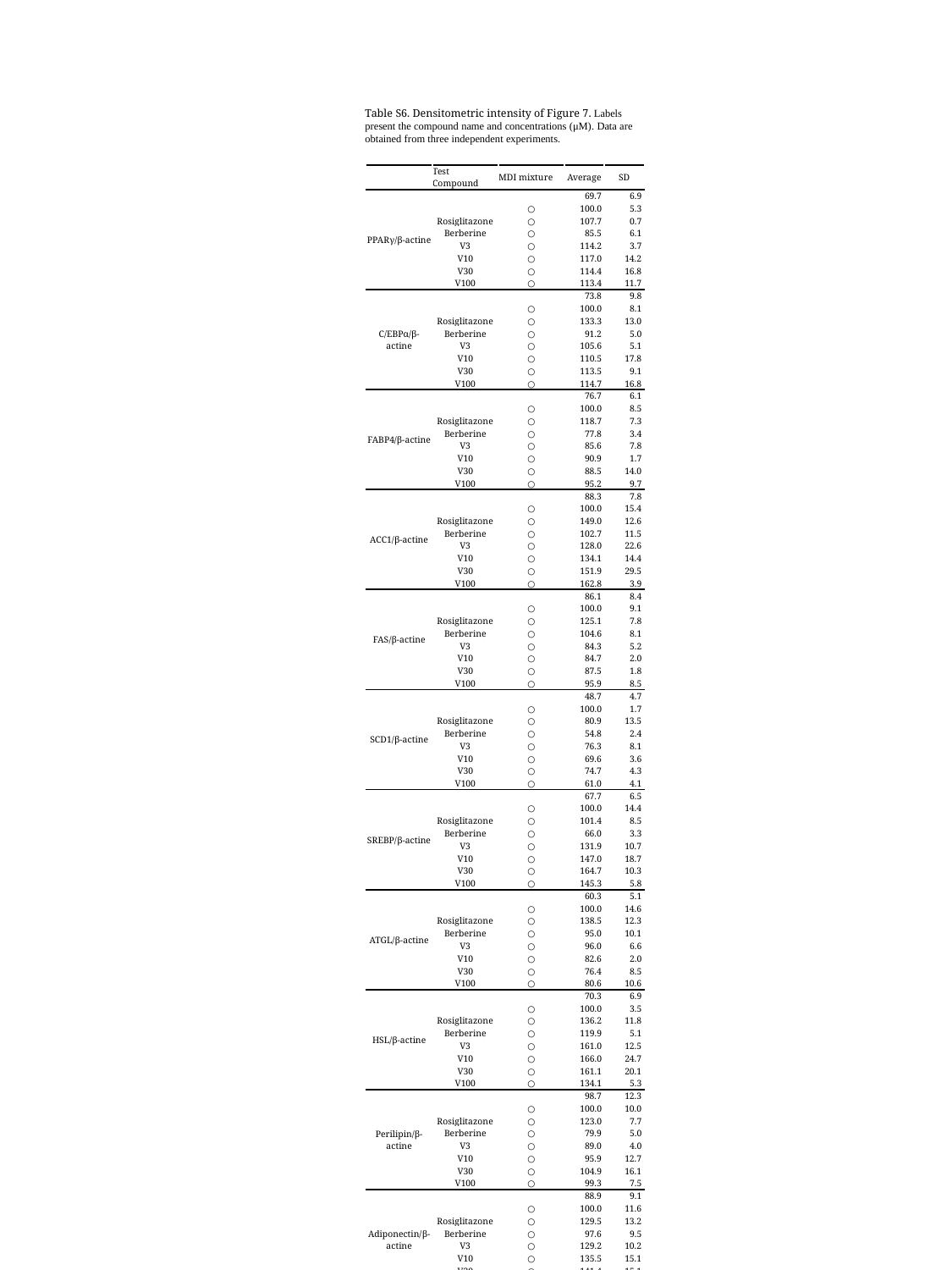

Table S6. Densitometric intensity of Figure 7. Labels present the compound name and concentrations (μM). Data are obtained from three independent experiments.
| | Test Compound | MDI mixture | Average | SD |
| --- | --- | --- | --- | --- |
| PPARγ/β-actine | | | 69.7 | 6.9 |
| | | ○ | 100.0 | 5.3 |
| | Rosiglitazone | ○ | 107.7 | 0.7 |
| | Berberine | ○ | 85.5 | 6.1 |
| | V3 | ○ | 114.2 | 3.7 |
| | V10 | ○ | 117.0 | 14.2 |
| | V30 | ○ | 114.4 | 16.8 |
| | V100 | ○ | 113.4 | 11.7 |
| C/EBPα/β-actine | | | 73.8 | 9.8 |
| | | ○ | 100.0 | 8.1 |
| | Rosiglitazone | ○ | 133.3 | 13.0 |
| | Berberine | ○ | 91.2 | 5.0 |
| | V3 | ○ | 105.6 | 5.1 |
| | V10 | ○ | 110.5 | 17.8 |
| | V30 | ○ | 113.5 | 9.1 |
| | V100 | ○ | 114.7 | 16.8 |
| FABP4/β-actine | | | 76.7 | 6.1 |
| | | ○ | 100.0 | 8.5 |
| | Rosiglitazone | ○ | 118.7 | 7.3 |
| | Berberine | ○ | 77.8 | 3.4 |
| | V3 | ○ | 85.6 | 7.8 |
| | V10 | ○ | 90.9 | 1.7 |
| | V30 | ○ | 88.5 | 14.0 |
| | V100 | ○ | 95.2 | 9.7 |
| ACC1/β-actine | | | 88.3 | 7.8 |
| | | ○ | 100.0 | 15.4 |
| | Rosiglitazone | ○ | 149.0 | 12.6 |
| | Berberine | ○ | 102.7 | 11.5 |
| | V3 | ○ | 128.0 | 22.6 |
| | V10 | ○ | 134.1 | 14.4 |
| | V30 | ○ | 151.9 | 29.5 |
| | V100 | ○ | 162.8 | 3.9 |
| FAS/β-actine | | | 86.1 | 8.4 |
| | | ○ | 100.0 | 9.1 |
| | Rosiglitazone | ○ | 125.1 | 7.8 |
| | Berberine | ○ | 104.6 | 8.1 |
| | V3 | ○ | 84.3 | 5.2 |
| | V10 | ○ | 84.7 | 2.0 |
| | V30 | ○ | 87.5 | 1.8 |
| | V100 | ○ | 95.9 | 8.5 |
| SCD1/β-actine | | | 48.7 | 4.7 |
| | | ○ | 100.0 | 1.7 |
| | Rosiglitazone | ○ | 80.9 | 13.5 |
| | Berberine | ○ | 54.8 | 2.4 |
| | V3 | ○ | 76.3 | 8.1 |
| | V10 | ○ | 69.6 | 3.6 |
| | V30 | ○ | 74.7 | 4.3 |
| | V100 | ○ | 61.0 | 4.1 |
| SREBP/β-actine | | | 67.7 | 6.5 |
| | | ○ | 100.0 | 14.4 |
| | Rosiglitazone | ○ | 101.4 | 8.5 |
| | Berberine | ○ | 66.0 | 3.3 |
| | V3 | ○ | 131.9 | 10.7 |
| | V10 | ○ | 147.0 | 18.7 |
| | V30 | ○ | 164.7 | 10.3 |
| | V100 | ○ | 145.3 | 5.8 |
| ATGL/β-actine | | | 60.3 | 5.1 |
| | | ○ | 100.0 | 14.6 |
| | Rosiglitazone | ○ | 138.5 | 12.3 |
| | Berberine | ○ | 95.0 | 10.1 |
| | V3 | ○ | 96.0 | 6.6 |
| | V10 | ○ | 82.6 | 2.0 |
| | V30 | ○ | 76.4 | 8.5 |
| | V100 | ○ | 80.6 | 10.6 |
| HSL/β-actine | | | 70.3 | 6.9 |
| | | ○ | 100.0 | 3.5 |
| | Rosiglitazone | ○ | 136.2 | 11.8 |
| | Berberine | ○ | 119.9 | 5.1 |
| | V3 | ○ | 161.0 | 12.5 |
| | V10 | ○ | 166.0 | 24.7 |
| | V30 | ○ | 161.1 | 20.1 |
| | V100 | ○ | 134.1 | 5.3 |
| Perilipin/β-actine | | | 98.7 | 12.3 |
| | | ○ | 100.0 | 10.0 |
| | Rosiglitazone | ○ | 123.0 | 7.7 |
| | Berberine | ○ | 79.9 | 5.0 |
| | V3 | ○ | 89.0 | 4.0 |
| | V10 | ○ | 95.9 | 12.7 |
| | V30 | ○ | 104.9 | 16.1 |
| | V100 | ○ | 99.3 | 7.5 |
| Adiponectin/β-actine | | | 88.9 | 9.1 |
| | | ○ | 100.0 | 11.6 |
| | Rosiglitazone | ○ | 129.5 | 13.2 |
| | Berberine | ○ | 97.6 | 9.5 |
| | V3 | ○ | 129.2 | 10.2 |
| | V10 | ○ | 135.5 | 15.1 |
| | V30 | ○ | 141.4 | 15.1 |
| | V100 | ○ | 146.7 | 14.5 |
| GLUT4/β-actine | | | 73.1 | 13.6 |
| | | ○ | 100.0 | 6.4 |
| | Rosiglitazone | ○ | 117.9 | 16.4 |
| | Berberine | ○ | 71.3 | 8.3 |
| | V3 | ○ | 75.5 | 7.5 |
| | V10 | ○ | 82.7 | 3.2 |
| | V30 | ○ | 97.1 | 5.2 |
| | V100 | ○ | 105.2 | 8.8 |
